# The Design of ZnO Nanorod Arrays Coated with MnOx for High Electrochemical Stability of a Pseudocapacitor Electrode

**DOI:** 10.3390/nano10030475

**Published:** 2020-03-06

**Authors:** Hsiang-Chun Chen, Yang-Ru Lyu, Alex Fang, Gang-Juan Lee, Lakshmanan Karuppasamy, Jerry J. Wu, Chung-Kwei Lin, Sambandam Anandan, Chin-Yi Chen

**Affiliations:** 1 Department of Materials Science and Engineering, Feng Chia University, Taichung 407, Taiwan; judy930410@hotmail.com (H.-C.C.); yrlyu.mse96g@g2.nctu.edu.tw (Y.-R.L.); 2 Department of Engineering Technology and Industrial Distribution, Texas A&M University, College Station, TX 77843, USA; gpafang@tamu.edu; 3 Department of Environmental Engineering and Science, Feng Chia University, Taichung 407, Taiwanlksamylaksh@gmail.com (L.K.); jjwu@mail.fcu.edu.tw (J.J.W.); 4 School of Dental Technology, College of Oral Medicine, Taipei Medical University, Taipei 110, Taiwan; 5 Research Center of Digital Oral Science and Technology, College of Oral Medicine, Taipei Medical University, Taipei 110, Taiwan; 6 Nanomaterials and Solar Energy Conversion Laboratory, Department of Chemistry, National Institute of Technology, Trichy 620015, India; sanand99@yahoo.com

**Keywords:** pseudocapacitor, ZnO nanorod, manganese oxide, chemical bath deposition, anodic deposition, cyclic voltammetry

## Abstract

Tremendous efforts have been made on the development of unique electrochemical capacitors or pseudocapacitors due to the overgrowing electrical energy demand. Here, the authors report a new and simple strategy for fabricating hybrid MnOx-coated ZnO nanorod arrays. First, the vertically aligned ZnO nanorods were prepared by chemical bath deposition (CBD) as a template providing a large surface area for active material deposition. The manganese oxide was subsequently coated onto the surface of the ZnO nanorods to form a hybrid MnOx-coated ZnO nanostructure by anodic deposition in a manganese acetate (MnA)-containing aqueous solution. The hybrid structure of MnOx-coated ZnO nanorod arrays exhibits a large surface area and high conductivity, essential for enhancing the faradaic processes across the interface and improving redox reactions at active MnOx sites. A certain concentration of the deposition solution was selected for the MnOx coating, which was studied as a function of deposition time. Cyclic voltammetry (CV) curves showed that the specific capacitance (SC) of the MnOx-coated ZnO nanostructure was 222 F/g for the deposition times at 10 s when the concentration of MnA solution was 0.25 M. The unique hybrid nanostructures also exhibit excellent cycling stability with >97.5% capacitance retention after 1200 CV cycles. The proposed simple and cost-effective method of fabricating hybrid nanostructures may pave the way for mass production of future intelligent and efficient electrochemical energy storage devices.

## 1. Introduction

Supercapacitors, also called electrochemical capacitors, are indispensable energy storage devices that have recently attracted great research interest from both academia and industry because they are specified to be as important as batteries for future energy storage systems. Also well-known is their usefulness in hybrid power applications requiring a high power output and/or a high cycle capacity, such as uninterruptible power supply units for computers, power electronics, and electric vehicles [1,2,3].

On the basis of their charge storage mechanisms, supercapacitors are generally classified into two main categories—electric double-layer capacitors (EDLCs) and pseudocapacitors [4,5]. EDLCs are composed of carbonaceous material such as activated carbon, carbon aerogel, etc., which have more specific surface area; unlike pseudocapacitors, which store electrical energy by the electrostatic accumulation of ionic charges in the electrical double-layer, nearby electrode, and electrolyte interfaces. Clearly, providing the material morphology with high specific surface area and enhancing its electrical conductivity are effective ways to obtain high specific capacitance in electrode material [6,7]. However, a pseudocapacitor commonly consists of a transition metal oxide such as RuO_2_ [8,9], MnO_2_ [10,11,12], V_2_O_5_ [13], or ZnO [14] possessing various valence states, which can store electronic or ionic charges not only by physical adsorption but also by reversible faradaic charge-transfer reaction occurring on the electrode surface. Theoretically, pseudocapacitors exhibit higher specific capacitance and energy density of more than 10 times as compared to EDLCs. Therefore, the pseudocapacitor has been considered to be a promising device for applications of high power density and high energy density due to their relatively fast and reversible faradic redox reactions.

**Figure 1:**
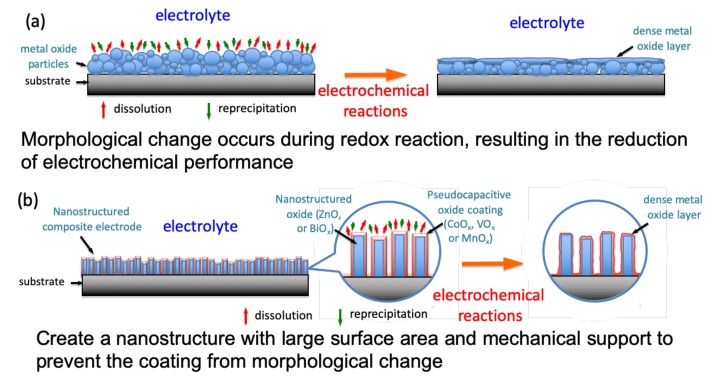
Schematic diagram of an electrochemical reaction between the surface of the pseudocapacitive material and the electrolyte solution. (**a**): Morphological change occurs during redox reaction, resulting in the reduction of electrochemical performance; (**b**): Creat a nanostructure with large surface area and mechanical support to prevent the coating from morphological change.

Transition metal oxides, Mn-oxide, and their compounds are being widely discovered for producing supercapacitors with increased specific capacitance and energy density because manganese possesses various oxidation states and versatile properties, is low in cost, has great flexibility in structure and morphology, is abundantly found in the earth, and is more environmentally friendly than other transition metal oxides [10,15,16,17,18]. Generally, the pseudocapacitive particles are coated onto substrates as an active material to react cyclically with the electrolyte solution. Such redox reactions, however, tend to change the microstructure of the active material by repeated dissolution and reprecipitation processes, as shown in Figure 1a. After a series of electrochemical reactions, the surface morphology of the powder coating thus becomes smoother with a reduction of exposed surface area to result in the decrease in specific capacitance. Therefore, we tried to design a nanostructure that provides large surface area and mechanical support for the active material while preventing change in surface morphology, as shown in Figure 1b. It is expected that the active material can still exhibit a large surface area and possess good electrochemical stability even after repeated faradic redox reactions.

Zinc oxide (ZnO), as one of the most attractive multifunctional materials, is extensively studied in piezotronics and electronic devices due to its excellent electric conduction and optoelectronic properties. Additionally, ZnO has strong mechanical support and high electron mobility as a result of its good chemical and thermal stabilities [19,20,21]. ZnO nanorod arrays (e.g., 70–900 nm in rod diameter) with an aspect ratio (rod length/rod diameter) of 5–20:1 can generally deliver a high specific surface area of 4–20 m^2^/g [22]. Hence, various approaches for synthesizing well-aligned ZnO nanorod arrays on different substrates have been extensively studied, such as chemical vapor deposition (CVD) [23,24], vapor-liquid-solid deposition [25,26], and solution-based growth methods [27,28,29,30]. Though the MnOx/ZnO composite has been demonstrated to exhibit a comparable SC value [31,32,33], the MnO_x_-coated ZnO nanostructure fabricated for supercapacitor applications has not yet been reported as a function of deposition condition. In the present study, vertically aligned ZnO nanorods were first synthesized by CBD as a template. Manganese oxide was subsequently coated onto the surface of ZnO nanorods to form a MnOx-coated ZnO nanostructure by anodic deposition as a function of deposition time. The microstructure, deposited morphology, and the corresponding electrochemical properties have been systematically investigated.

## 2. Materials and Methods

### 2.1. Materials

The following compounds were used: Zn(CH_3_COO)_2_∙4H_2_O (Showa, Japan), Zn(NO_3_)_2_·4H_2_O (J. T. Baker, Phillipsburg, NJ, USA), C_6_H_12_N_4_ (Alfa Aesar, Ward Hill, MA, USA) and Mn(CH_3_COO)_2_∙4H_2_O (Acros Organics, Carlsbad, CA, USA) and further abbreviated in this report as ZnA, ZnN, HMT, and MnA, respectively. All the chemicals used in this study are reagent grade with higher than 99.0% purity.

### 2.2. Materials Preparation

The zinc oxide nanorod with a vertically aligned orientation was first synthesized with various deposition times on stainless steel substrate (10 × 10 × 1.2 mm^3^) as a template for the deposition of MnOx by chemical bath deposition [34,35]. Prior to deposition, the stainless-steel substrate was ultrasonically cleaned in acetone, isopropyl alcohol, ethanol, and deionized water for 10 min, respectively, and dried at room temperature in air. Similar to the process in the literature [34], a solution of a 0.5 mM zinc acetate (ZnA) dissolved in ethanol was drop-coated onto the pre-treated substrate and then heat-treated at 300 °C for 30 min (HTF55322C, Thermo Scientific Lindberg/Blue M, Asheville, NC) as a seed layer for ZnO nanostructure deposition. During the deposition process, a solution of 0.1 M zinc nitrate (ZnN) dissolved in hexamethylenetetramine (HMT) was prepared as a zinc source. After stirring at room temperature, ammonium hydroxide was added to the solution to adjust the pH = 6. The ZnO nanorods were grown by submerging the pre-seeded substrate into the zinc-containing solution at a bath temperature of 70 °C for 3–6 h. This process gave a total amount of ZnO nanorod ca. 2–4 mg/cm^2^. As a template for MnOx deposition, the ZnO nanorod-deposited substrates were subsequently rinsed with deionized water and dried at room temperature in air.

Pseudocapacitive manganese oxide was deposited onto the surface of the ZnO nanorods in an aqueous manganese acetate (MnA) solution by anodic deposition (AD) for deposition times of 10, 20, 30, and 40 s. The concentration of MnA solution was 0.25 M. The anodic potential was set at 0.5 V. Subsequently, the MnOx-coated ZnO nanostructured electrode was obtained by drying at room temperature in air.

### 2.3. Characterizations

The morphology examination and nanostructural analysis of the resulting core-shell electrodes were performed using scanning electron microscopy (SEM, JSM-6700F, JEOL, Tokyo, Japan) and transmission electron microscopy (TEM, 2100F, JEOL II, Tokyo, Japan), respectively. The phase identification was carried out by X-ray diffractometry (XRD, SRA-M18XHF, MAC Science, Yokohama, Japan). The elemental and chemical compositions were determined by energy dispersive X-ray spectroscopy (EDS, attached to JEOL JSM-6700F) and X-ray photoelectron spectroscopy (XPS, PHI 5000 VersaProbe, ULVAC-PHI, Chigasaki, Japan), respectively. Electrochemical characterization of the electrodes was performed in a three-compartment cell by an electrochemical analyzer system (Model 727C, CH Instruments, Austin, TX, USA) at room temperature. An Ag/AgCl electrode was used as the reference electrode, and a piece of platinum foil served as the counter electrode. The morphologies of the Mn/Zn-oxide coatings before and after CV were observed using scanning electron microscopy (SEM, JSM-6700F, JEOL, Tokyo, Japan).

## 3. Results and Discussions

**Figure 2:**
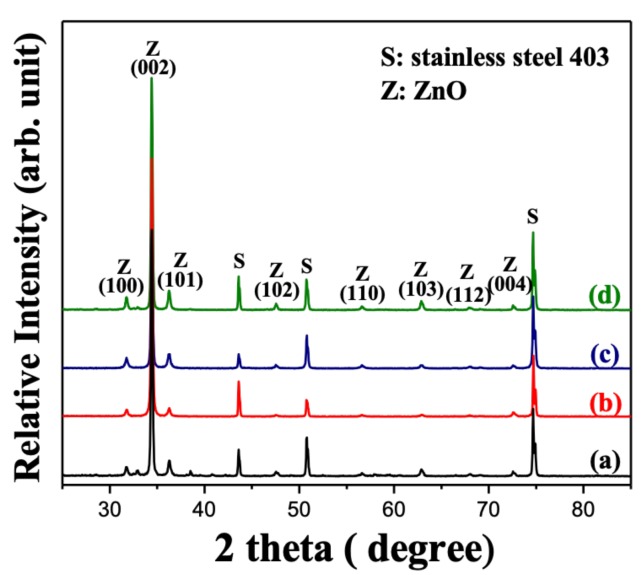
X-ray diffraction (XRD) patterns of the ZnO nanorods deposited under growth condition of pH = 6 by chemical bath deposition at 70 °C for (**a**) 3, (**b**) 4, (**c**) 5, and (**d**) 6 h.

**Figure 3:**
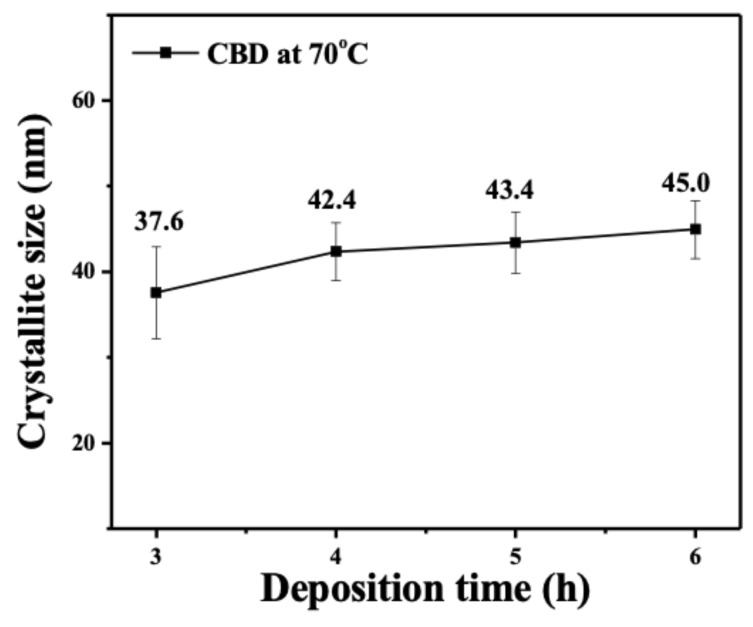
Crystallite size of the ZnO nanorods deposited under growth condition of pH = 6 by chemical bath deposition (CBD) at 70 °C as a function of deposition time.

The growth of the ZnO nanostructure on the stainless-steel substrate by the chemical bath deposition (CBD) method generally depends on the deposition time, temperature and pH condition. The X-ray diffraction patterns were utilized to confirm the crystalline structure of fabricated ZnO/stainless steel composite by CBD, as shown in Figure 2. The deposited ZnO nanostructure on stainless steel substrate as a function of various deposition time exhibited sharp and clear diffraction peaks, which are well indexed based on crystalline hexagonal wurtzite phase of ZnO (JCPDS card no.: 80-0075). The strong diffraction peak of (002) planar indicated that the ZnO nanostructure appeared with the preferred hexagonal orientation of the *c*-axis. The heterogeneous growth of the ZnO nanostructure was enhanced with the increase in deposition time. The crystallinity was further estimated using the Scherrer equation. Figure 3 shows the crystallite size of ZnO nanostructures as a function of various deposition time. Note that the crystallite size increased as the deposition time increased, suggesting that the enhancement in the crystalline growth of the preferred orientation of the ZnO nanostructure corresponds with prolonging the deposition time.

**Figure 4:**
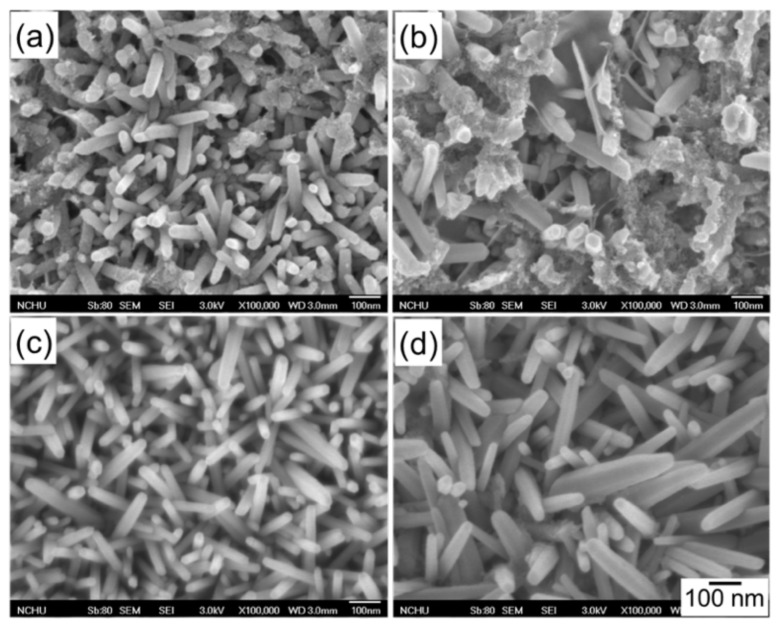
Scanning electron microscopy (SEM) micrographs of the ZnO nanorods deposited under growth condition of pH = 6 by CBD at 70 °C for deposition times of (**a**) 3, (**b**) 4, (**c**) 5, and (**d**) 6 h.

Figure 4a–d shows the top-view SEM images of the bare ZnO nanostructure via CBD at 70 °C for various deposition times. From the micrograph image, the ZnO nanostructure revealed randomly orientated nanorods with the preferred vertical orientation, which agrees with the results of XRD as shown in Figure 2. Furthermore, it was observed that the dimension of the ZnO nanorods increased with the increase in deposition time. Though not shown here yet, the ZnO nanorod after 6 h of deposition exhibited better capacitive properties; thus, the 6 h grown ZnO template was employed for the anodic deposition of the pseudocapacitive MnOx.

**Figure 5:**
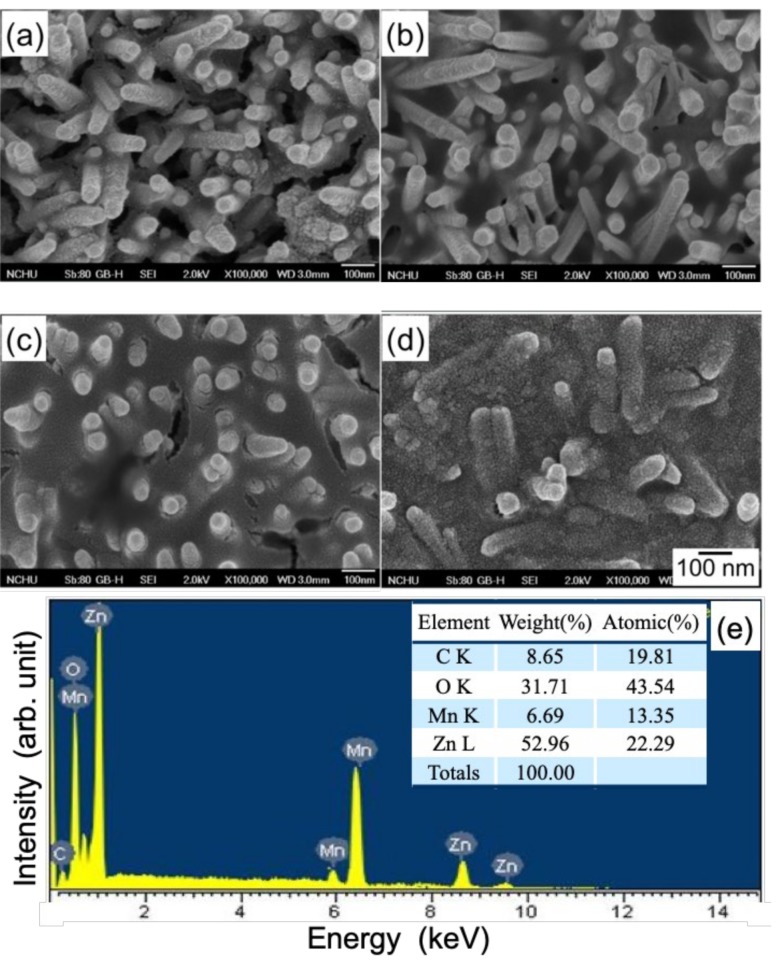
SEM micrographs of the MnOx-coated ZnO nanorod for MnOx deposition times of (**a**) 10, (**b**) 20, (**c**) 30, and (**d**) 40 s. (**e**) shows the EDX spectrum and elemental composition of the MnOx-coated ZnO nanorod at a deposition time of 10 s. The MnOx was coated by anodic deposition in a 0.25 M Mn(CH_3_COO)_2_·4H_2_O solution at 25 °C.

Figure 5 shows the top-view SEM micrographs of the MnOx-coated ZnO nanorod as a function of MnOx deposition time. Note that the amount of MnOx coating increased with the increase in deposition time, and almost covered the ZnO nanorods after 40 s of anodic deposition (AD). However, in Figure 5a,b, the AD MnOx coating is imperceptible in the 10 s and 20 s deposited samples in the SEM images. Although not shown here, even after 40 s of MnOx deposition, the resolution of XRD is also unable to reveal the presence of MnOx on ZnO nanorods due to the small amount of coating. Therefore, EDX and XPS (to be discussed in Figure 11) spectra were further carried out for the elemental and oxidation state analyses, respectively, to confirm the MnOx deposition. Figure 5e shows respectively the EDX spectrum and elemental composition of the MnOx-deposited ZnO nanorod at a deposition time of 10 s. Manganese signals were detected in the MnOx-coated ZnO nanorod after just 10 s of deposition, revealing that the MnOx can be coated onto the surface of the ZnO nanorods in a relatively short deposition time.

**Figure 6:**
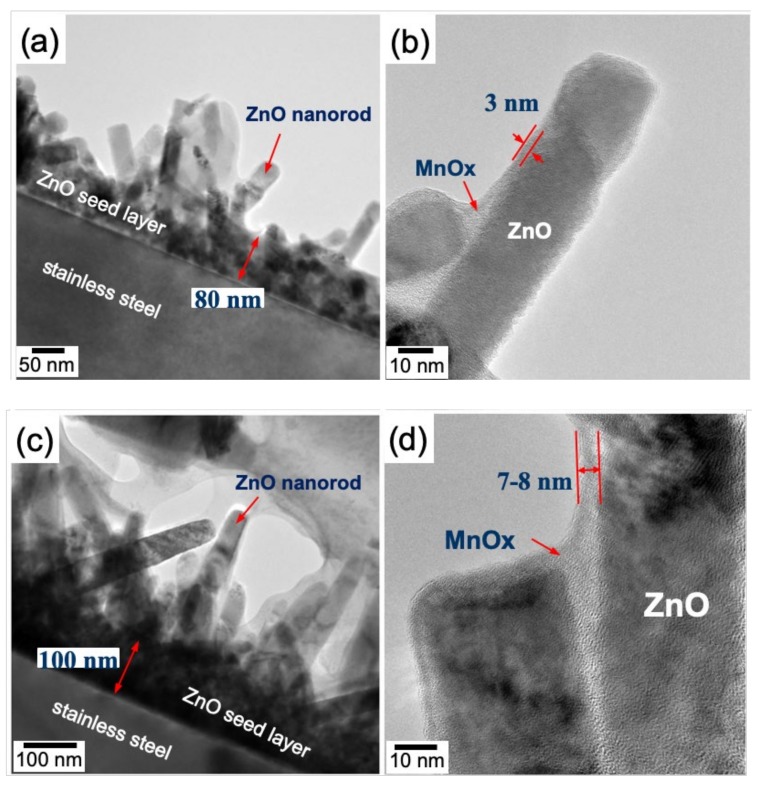
Cross-sectional TEM micrographs of the MnOx-deposited ZnO nanorod at deposition times of (**a**,**b**) 10 s and (**c**,**d**) 20 s. The MnOx was coated by anodic deposition in a 0.25 M Mn(CH_3_COO)_2_·4H_2_O solution at 25 °C.

To further reveal the details of the MnOx deposition, the morphology of the MnOx-coated ZnO nanorod, as well as the stainless-steel substrate, was confirmed by TEM observation in the present study. Figure 6 shows the cross-sectional TEM micrographs of ZnO nanorods with MnOx coatings after MnOx deposition, with different deposition times. Such advanced electron microscopy related techniques provide an effective route for examining the coverage uniformity and thickness of the AD MnOx coating, as well as the interface between the MnOx coating and the ZnO template. Note that the seed layer consisted of randomly distributed ZnO nanocrystallites with a thickness of ca. 80–100 nm (Figure 6a,c). The CBD ZnO nanorods with ca. 20–45 nm in diameter were then revealed to grow in a spatially confined mode due to the high density of seeds, forcing a vertical alignment during nanorod formation [36]. Moreover, a translucent thin layer was observed on the surface of the ZnO nanorods, suggesting an extremely poor crystalline AD MnOx coating. Almost all the surfaces of the ZnO nanorod were covered with a relatively uniform MnOx coating. The thicknesses of the 10s and 20s deposited MnOx coatings were, respectively, ca. 3–4 nm and 7–8 nm. Figure 6b,d shows an increasing trend with prolonging the AD time. Indeed, these results are in agreement with the SEM results.

**Figure 7:**
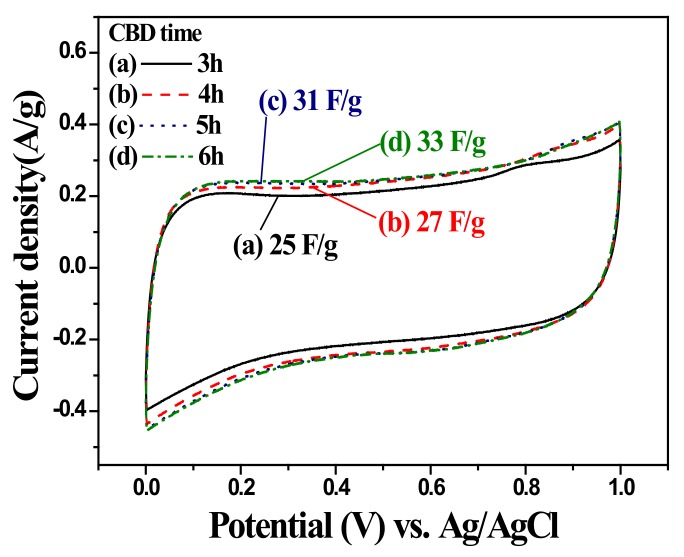
Cyclic voltammograms of ZnO nanorod measured from the 50th cycle in 1.0 M Na_2_SO_4_ electrolyte at a scan rate of 25 mV/s as a function of CBD time. The ZnO nanorods were deposited under growth condition of pH = 6 by CBD at 70 °C.

**Figure 8:**
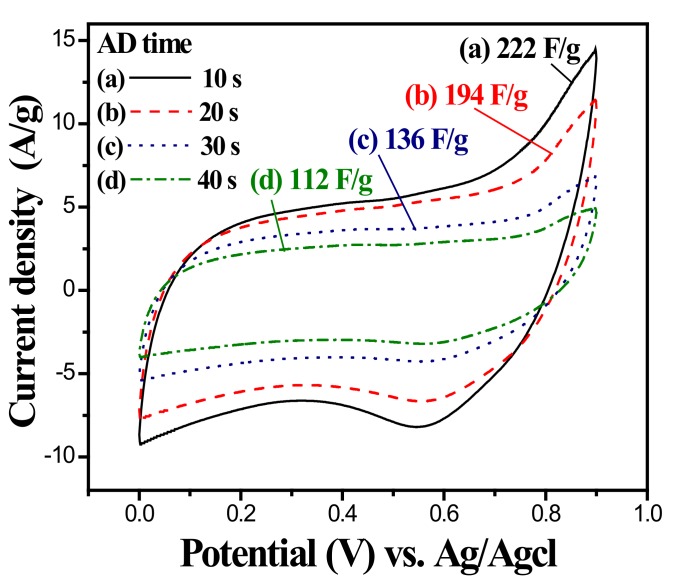
Cyclic voltammograms of MnOx-coated ZnO nanorod measured from the 50th cycle in 1.0 M Na_2_SO_4_ electrolyte at a scan rate of 25 mV/s as a function of anodic deposition time. The MnOx was coated in a 0.25 M Mn(CH_3_COO)_2_·4H_2_O solution at 25 °C.

The pseudocapacitive characteristics of the ZnO nanorod with and without MnOx coating were analyzed by cyclic voltammetry and life tests. Figure 7 shows CV curves of the uncoated ZnO nanorod in 1.0 M Na_2_SO_4_ in the range of 0 to 1.0 V with a scan rate of 25 mV/s as a function of CBD growth time before the preparation of MnOx-coated equivalent. Note that the CV curves were almost rectangular in shape, revealing a typical capacitive behavior with a charging current in both scanning directions across the potential range of 0 to 1.0 V. The CV curves demonstrated that the uncoated ZnO nanorods had good redox reversibility. Moreover, the area under the CV curve can be used to estimate the specific capacitance (SC) of the system according to the following equations [37,38,39,40]:

(1) $$C=\frac{1}{\Delta V\left(dV/dt\right)}\int IdV$$

(2) $$C_{sp}=\frac{A}{\Delta V\times v\times m}$$

where *C* and *C_sp_* are the integral capacitance and specific capacitance of the electrode, respectively. *dV*/*dt* is the voltage scan rate, *I* is the voltammetric current, *A* is the integral area of the cyclic voltammogram loop, Δ*V* is the sweep potential window, *v* is the scan rate, and *m* is the mass of the active materials at the electrode. The SCs of the uncoated ZnO nanorod were measured from the 50th CV tests. The SCs of the uncoated ZnO nanorods were 25, 27, 31, and 33 F/g for CBD grow times of 3, 4, 5, and 6 h, respectively. Though the ZnO nanorods exhibited capacitive behaviors, the SCs were still relatively low in comparison with those of pseudocapacitive materials, such as RuO_2_, MnO_2_, V_2_O_5_ [10,11,12,13]. The 6 h grown ZnO nanorod possessing the highest SC value was used as the template for the MnOx deposition. Figure 8 shows CV curves of the MnOx-coated ZnO nanorod in 1.0 M Na_2_SO_4_ in the range of 0 to 1.0 V with a scan rate of 25 mV/s as a function of MnOx AD times. Similarly, the MnOx-coated samples having almost rectangular CV curves revealed a typical capacitive behavior and a good redox reversibility. Note that the SCs of the MnOx-coated ZnO nanorods were estimated to be 222, 194, 136, and 112 F/g for the deposition times of 10, 20, 30 and 40 s, respectively. The SC decreased with the increase of MnOx AD time. That is, the thicker MnOx coating may result in the decrease of specific capacitance in this system. An appropriate thickness of MnOx coating was thought to provide a more effective surface area for faradaic redox reaction, thus elevating the specific capacitance of the active material.

**Figure 9:**
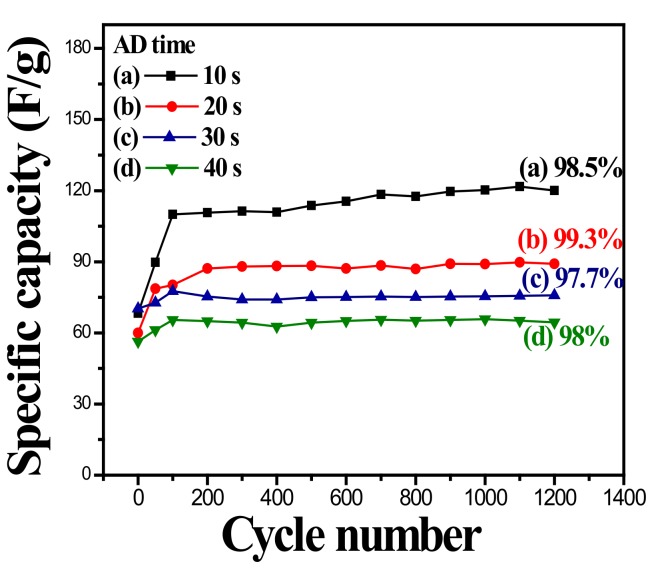
Specific capacitance of MnOx-coated ZnO nanorod measured in 1.0 M Na_2_SO_4_ electrolyte at a scan rate of 100 mV/s at various anodic deposition time as a function of cycle number.

The electrochemical stability of the MnOx-coated ZnO nanorod was investigated by repeating the CV test at a scan rate of 100 mV/s for 1200 cycles. Figure 9 shows the long-term cycle performance of the MnOx-coated ZnO nanorod in 1.0 M Na_2_SO_4_ electrolyte as a function of MnOx AD time. Because the slow diffusion rate of electrolyte ions gains access to the available sites by intercalation and/or absorption [41], the higher the scan rate, the lower the measured SC (comparing to the data in Figure 8). The SC of all the samples increases dramatically before 100 cycles of CV, implying that the surface of the coatings was activated at the initial stage. An imperceptible decline in SC of the samples with increased CV cycle number can be observed after 200 cycles. That is, the capacitance of the MnOx-coated ZnO nanorod tended to decline slightly after the activation of the initial 200 cycles until the cycle number increased to 1200. The cycling efficiencies (minimum/the maximum SC ratio) of the samples in the 1200-cycle life tests are also indicated in the figure. In the literature, without template/scaffold supports, MnOx coatings tend to change the surface morphology into denser and smoother structures by the adsorption/desorption or the intercalation/de-intercalation process, causing a decline in SC during the repeated charging/discharging process [42]. As shown in the figure, the data indicates that the ZnO nanorod can significantly improve the cycling stability of the MnOx coatings up to an efficiency higher than 97.5%. The MnOx-coated ZnO nanorods possess relatively good efficiencies with a good electrochemical performance for supercapacitors. It is believed that the ZnO nanorod provided a template for avoiding the significant change in surface morphology of the MnOx coating after long-term repetitive cycling tests, resulting in high electrochemical stability.

**Figure 10:**
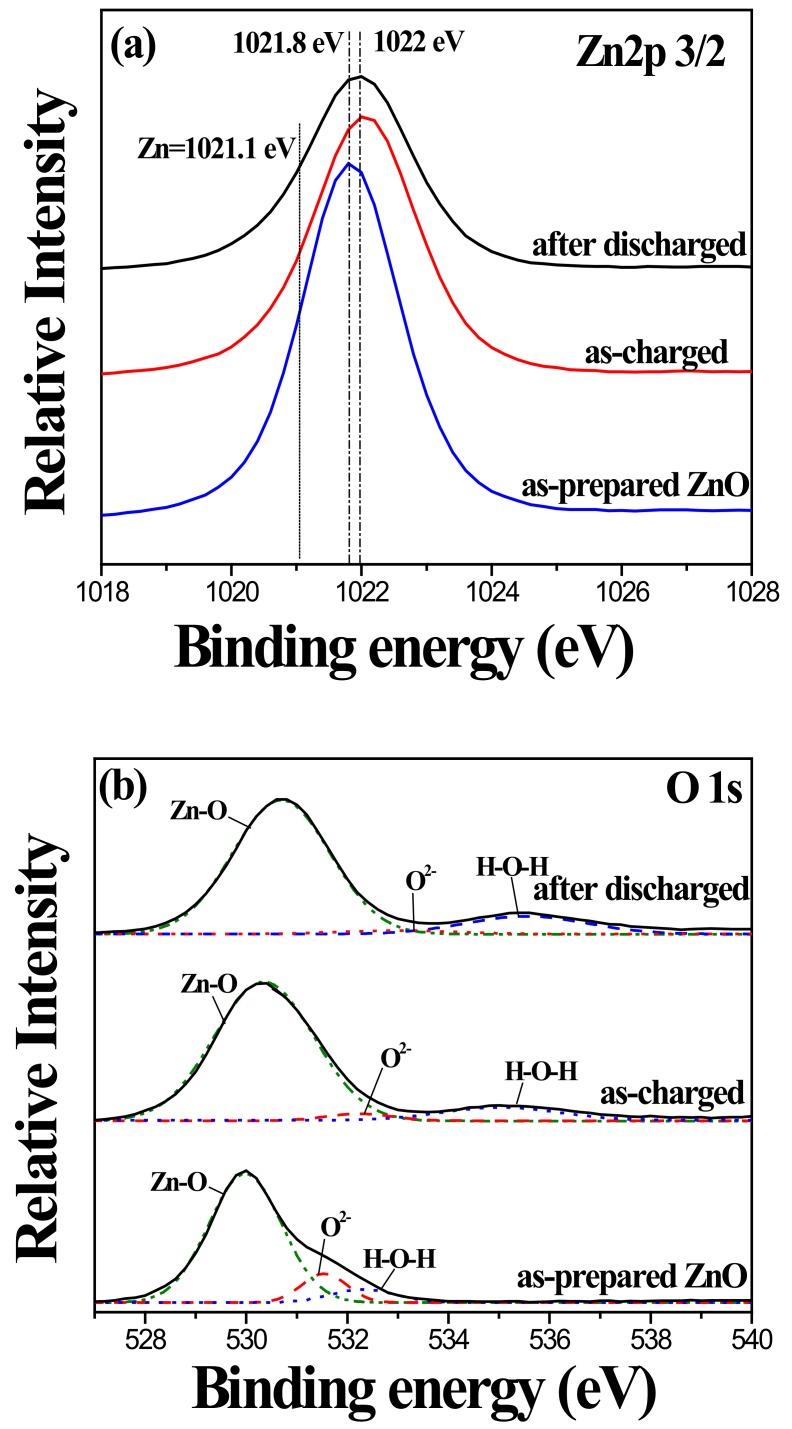
(**a**) Zn 2p 3/2 and (**b**) O 1s XPS spectra of uncoated ZnO nanorod before and after CV test.

**Figure 11:**
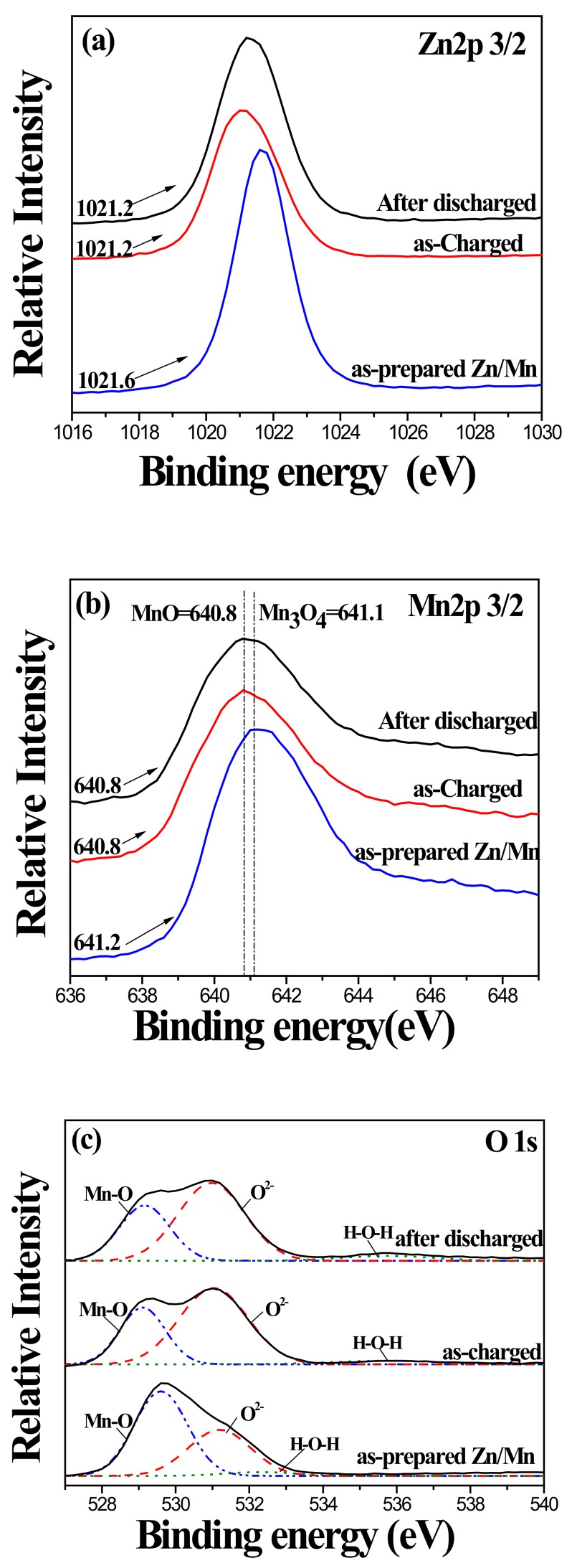
(**a**) Zn 2p 3/2, (**b**) Mn 2p 3/2 and (**c**) O 1s XPS spectra of MnOx-coated ZnO nanorod before and after CV test.

To clarify the role of the ZnO nanorod during the electrochemical reactions in this system, the changes of electronic structures or chemical valence state of each element of the ZnO template with and without MnOx coating before and after charging reactions were carried out by XPS analyses. Figure 10 shows the Zn 2p3/2 and O 1s XPS spectra of the uncoated ZnO nanorod before and after the CV test. As seen in Figure 10a, the binding energy of the Zn 2p3/2 peak was slightly shifted from 1021.8 eV to 1022.0 eV after the charging reaction. Such a small change in the oxidation state of Zn^2+^ in ZnO, an almost negligible change during a faradaic redox reaction, implies that the ZnO nanorod has low specific capacitance. On the other hand, the high-resolution O 1s spectrum (Figure 10b) can be deconvoluted into two peaks of the uncoated Zn-O bond (530.2 eV) and H-O-H bond (surface-adsorbed water, 532.6 eV). As the result suggests, the presence of adsorbed H_2_O and O_2_ on the surface of the ZnO nanorod is highly inhibitive of SC. Figure 11 shows the Zn 2p3/2, Mn 2p3/2, and O 1s XPS spectra of the MnOx-coated ZnO nanorod before and after the CV test. As shown in Figure 11a, the shift in the Zn 2p3/2 peak from 1021.8 eV to 1021.2 eV is associated with the change of Zn(+2) to Zn(0), which indicates that the MnOx coating may reduce nanorod ZnO to Zn during repetitive CV cycles. Moreover, the Mn 2p3/2 peak at a binding energy of 641.2 eV and 640.8 eV, Figure 11b, of the Mn-coated ZnO nanorod, were addressed as oxidation states of +2.66 (Mn_3_O_4_) and +2 (MnO), respectively. This suggests that the MnOx coating changed from Mn_3_O_4_ to MnO after repetitive charging/discharging reactions. As reported in the literature, pseudocapacitive Mn_3_O_4_ are generally expected to react to MnO_2_ after experiencing a series of CV tests [12]. In the case of the MnOx coating on the ZnO nanorod, the reaction of the MnOx coating from Mn_3_O_4_ to MnO was influenced by the combination of the pseudocapacitive reaction with the electrolyte solution and the redox reaction with the ZnO nanorod. Such interactions may cause the limited specific capacitance of the MnOx coating. In the present study, however, the design of the pseudocapacitor electrode based on MnOx-coated ZnO nanostructured material has been proposed for a promising solution for the applications of a supercapacitor with high electrochemical stability.

## 4. Conclusions

The MnOx-Coated ZnO nanostructure was successfully prepared via a combination of chemical bath deposition and anodic deposition for use as a potential pseudocapacitor electrode material with excellent electrochemical stability. As a template for pseudocapacitive MnOx coating, vertically grown wurtzite ZnO nanorods on a stainless-steel substrate provided an efficiently mechanical scaffold for the electrochemical reaction of active materials. The AD MnOx coating with 3–8 nm in thickness and a good coverage exhibited an acceptable specific capacitance of 222 F/g for the deposition times of 10 s. Furthermore, the nanostructural design of the pseudocapacitor electrode based on MnOx-coated ZnO material possessed better behavior of electrochemical energy storage, with higher cycling efficiencies of better than 97.5% after 1200 CV cycles and can be considered as a highly promising candidate for a pseudocapacitor.
